# Robust computer-vision based construction site detection for assistive-technology applications

**DOI:** 10.1080/17483107.2026.2618130

**Published:** 2026-02-01

**Authors:** Junchi Feng, Giles Hamilton-Fletcher, Nikhil Ballem, Michael Batavia, Yifei Wang, Jiuling Zhong, Mahya Beheshti, Maurizio Porfiri, John-Ross Rizzo

**Affiliations:** a Department of Biomedical Engineering, Tandon School of Engineering, New York University, Brooklyn, NY, USA; b Center for Urban Science and Progress, Tandon School of Engineering, New York University, Brooklyn, NY, USA; c Department of Ophthalmology, NYU Grossman School of Medicine, New York, NY, USA; d Department of Rehabilitation Medicine, NYU Grossman School of Medicine, New York, NY, USA; e Department of Mechanical and Aerospace Engineering, Tandon School of Engineering, New York University, Brooklyn, NY, USA

**Keywords:** Assistive technology, blindness and low vision, construction site, disability, open-vocabulary object detection, optical character recognition

## Abstract

**Purpose:**

Navigating urban environments poses significant challenges for individuals who are blind or have low vision, especially in areas affected by construction. Construction zones introduce hazards such as uneven surfaces, barriers, hazardous materials, excessive noise, and altered routes that obstruct familiar paths and compromise safety. Although navigation tools assist in trip planning, they often overlook these temporary obstacles. Existing hazard detection systems also struggle with the visual variability of construction sites.

**Methods:**

We developed a computer vision–based assistive system integrating three modules: an open-vocabulary object detector to identify diverse construction-related elements, a YOLO-based model specialised in detecting scaffolding and poles, and an optical character recognition module to interpret construction signage.

**Results:**

In static testing at seven construction sites using images from multiple stationary viewpoints, the system achieved 88.56% overall accuracy. It consistently identified relevant objects within 2–10 m and at approach angles up to 75°. At 2–4 m, detection was perfect (100%) across all angles. Even at 10 m, six of seven sites remained detectable within a 15° approach. In dynamic testing along a 0.5-mile urban route containing eight construction sites, the system analysed every frame of a first-person walking video. It achieved 87.26% accuracy in distinguishing construction from non-construction areas, rising to 92.0% with a 50-frame majority vote filter.

**Conclusion:**

The system can reliably detect construction sites in real time and at sufficient distances to provide advance warnings, enabling individuals with visual impairments to make safer mobility decisions, such as proceeding with caution or rerouting.

## Introduction

Metropolitan areas are home to nearly two-thirds of adults with disabilities in the United States, making urban accessibility a crucial public concern [1]. While many cities provide extensive public transit and increasingly inclusive infrastructure, significant barriers to independent mobility persist for people who are blind or have low vision (pBLV). Among these challenges, the “first-and-last-mile” problem—navigating the often short but unpredictable distances to and from transit stops—remains a persistent obstacle. On this segment of the journey, unpredictable street furniture, parked vehicles, inaccessible crossings, and especially hazardous construction sites can disrupt navigation and threaten safety [2–4].

Construction zones, an ever-present feature of growing urban areas [5], are especially problematic for pBLV. In New York City alone, on an average day, there may be more than 1100 active construction sites and over 9000 sidewalk sheds that span an incredible 400 miles of sidewalk [6,7]. These sites introduce temporary and unpredictable hazards—uneven ground, barriers, noisy equipment, and blocked paths—that disrupt spatial memory, mask auditory cues, and force sudden detours [8–10]. For sighted pedestrians, construction obstacles are often noticed from a distance and avoided based on visual cues. In contrast, pBLV rely primarily on memorised routes, tactile feedback, and environmental sounds—all of which are easily disrupted by new or unannounced construction activity. The consequences of encountering an unexpected construction site can be serious. Without timely awareness, pBLV risk physical injury, disorientation, or being stranded at an impassable section of sidewalk. Often, the only recourse is to seek help from strangers or attempt risky detours, directly undermining both safety and independence. Even a brief or localised obstruction can have a major impact, forcing users to cross busy streets, backtrack long distances, or abandon their planned route.

Despite the prevalence and impact of these hazards, existing navigation aids offer little real-time protection. Mainstream trip-planning apps like Google Maps provide information on major road closures or transit disruptions, but rarely account for smaller, temporary construction projects that block pedestrian pathways [11,12]. Research on computer vision-based detection has made progress, but typical approaches rely on convolutional neural networks (CNNs) trained on fixed, annotated datasets [13–15]. Such systems struggle with the variability and novelty of construction objects, which differ between cities and are constantly changing [16]. Furthermore, much of the existing work focuses on monitoring construction activity through surveillance video, not on providing actionable, real-time alerts to pedestrians [17,18]. Our work is motivated by the need for in-the-moment hazard detection and immediate, practical decision support for pBLV. Since construction sites often appear unexpectedly and may not be mapped in advance, a real-time warning system is essential. We do not propose to automatically reroute users or alter their entire journey in advance. Instead, our goal is to empower pBLV to make safer, more informed decisions at critical moments: for example, choosing to cross at a controlled intersection before encountering a blocked sidewalk, turning back before reaching a dead end, or waiting safely for assistance rather than attempting an unsafe detour. By delivering timely alerts just before important decision points, our system directly supports safe, independent navigation—enabling users to avoid sudden hazards, minimise disorientation, and retain control of their travel.

To address this need, we propose a novel open-vocabulary, computer vision-based construction site detection system designed specifically for pedestrian use. Our model leverages open-vocabulary detection to recognise and localise a wide range of construction- related objects—including tools, barriers, machinery, and signage—using textual prompts rather than a fixed set of categories. We further supplement this with a YOLO-based model trained on key classes and an optical character recognition (OCR) module for identifying construction signage, ensuring broad coverage and real-time performance. This hybrid approach reduces dependence on limited datasets, adapts to new and unfamiliar construction environments, and, most importantly, delivers actionable alerts that support real-time, proactive navigation decisions for pBLV. Our hypothesis was that integrating open-vocabulary detection and OCR would improve the accuracy, reliability, and practical value of construction site detection in dynamic urban settings. We validate this hypothesis through experiments that measure detection accuracy, angular offset, and effective range under realistic field conditions.

The objective of this study was to develop and evaluate a computer vision-based system for real-time construction site detection to better support assistive navigation for pBLV. We hypothesised that accurate detection cannot be achieved using a single detection module due to the diverse appearance of construction elements and the visual similarity of unrelated urban objects. We predicted that a modular system combining open-vocabulary object detection, a YOLO-based scaffolding detector, and an OCR-based sign recogniser would yield more accurate and robust performance.

## Methods

### The detection of construction sites

A single construction site-related object is insufficient to confirm the presence of a construction site. We developed a comprehensive decision-making framework to afford the reliable inference of construction zones. This framework consists of three pipelines, each analysing the image from a different perspective: sidewalk scaffolding, construction site-related objects, and construction signs.

#### Sidewalk scaffolding detection

A commonly encountered construction feature in New York City is sidewalk scaffolding—a temporary structure installed above sidewalks to protect pedestrians from falling debris during building construction or renovation [19]. Sidewalk scaffolding typically consists of three main components: decking, vertical supports, and bracing [20]. The decking forms the overhead platform and is usually made of wooden or steel planks. From a pedestrian’s perspective, it appears as a flat, uniformly coloured surface—often dark green or brown—with little texture or edge contrast. As a result, object detection models struggle to identify the decking directly. The vertical supports are upright steel poles that hold up the decking, while bracing refers to diagonal or horizontal elements that stabilise the structure and prevent collapse. These components are also visually challenging to detect, as they often resemble other vertical or linear features in urban environments—such as traffic light poles, streetlamps, or window frames. Despite this ambiguity, vertical supports and bracing offer more consistent geometric patterns and contrast than the flat decking and are therefore more suitable as detection targets.

Our model identifies the presence of sidewalk scaffolding by detecting vertical supports and horizontal bracing, typically steel poles in NYC [20]. Theoretically, a sidewalk shed requires a minimum of four steel poles to form a stable rectangular frame (two on each side) and two diagonal braces to prevent swaying, resulting in at least six steel poles. To allow for some tolerance for false negatives, detecting at least five steel poles—either vertical supports or bracing—confirms the presence of a construction site.

#### Construction-related objects detection

The presence of construction-related objects, such as traffic barricades, barrels, cones, green wooden walls, and similar items, is often strong evidence of an active construction site. These objects secure the area, guide pedestrians, and facilitate construction activities. In most cases, construction sites contain multiple construction-related objects to meet essential safety and operational requirements. Therefore, detecting at least three construction-related objects serves as a criterion for confirming a construction site. This threshold reflects the typical presence of multiple objects at construction sites while allowing some tolerance for false positives triggered by some random objects on the street.

#### Construction-sign detection

Signage serves as strong evidence of a construction site. Construction signage refers to an informational sign used to warn, guide, or manage traffic in or around a construction zone. Such signs are difficult for pBLV to localise and interpret. A sign explicitly displaying text such as “Construction Zone” is sufficient to confirm the presence of a construction site. Therefore, the detection of a single sign typically used in construction sites is enough to conclude the existence of a construction site.

#### Hybrid combinations of detections

Each detection pipeline (scaffolding, construction-related objects, and signage) has its own threshold for confirming a construction site. However, real-world conditions such as occlusion, lighting variability, and partial views may prevent any single pipeline from fully meeting its criterion. It is also common for construction sites to include both sidewalk scaffolding and elements like traffic barricades or green walls to protect pedestrians. To address this, we implemented a hybrid decision rule that combines detections from the scaffolding and object pipelines to jointly infer the presence of a construction site.

The hybrid threshold is set to a total of five detected elements, where each element is either a scaffolding pole or a construction-related object. This value is motivated by the individual thresholds: the scaffolding detector requires five poles, and the object detector requires three objects. The hybrid rule assumes that a co-occurrence of fewer elements from each category, such as three poles and two objects, can collectively offer strong contextual evidence. Combinations below this threshold are considered too weak and more likely to appear in non-construction environments, which increases the risk of false positives. The threshold of five was empirically selected based on exploratory analysis of labelled training data to balance sensitivity with specificity.

### Sidewalk scaffolding detection

As previously mentioned, our model identifies the presence of sidewalk scaffolding by detecting vertical supports and horizontal or diagonal bracing, typically made of steel poles. However, detecting sidewalk scaffolding poles using an open-vocabulary approach is particularly challenging due to their visual similarity to other vertical structures commonly found in urban environments, such as streetlight poles, glass window frames, and traffic sign poles.

To address this challenge, we trained a YOLOv8 model specifically designed to detect sidewalk scaffolding poles. Volunteers collected images of sidewalk scaffolding, emphasising scaffolding poles as the primary structural elements. This effort resulted in a comprehensive dataset of 447 images captured in diverse urban settings and under varying lighting conditions to ensure broad representation for training. The dataset was divided into training and validation subsets in an 80/20 split. Two distinct classes were annotated: scaffolding poles, which are vertical poles essential for supporting sidewalk sheds, and horizontal scaffolding, which includes poles oriented horizontally or nearly horizontally to connect vertical poles.

In total, 2297 annotations were made for horizontal scaffolding and 2593 annotations for scaffolding poles, providing a solid foundation for model training. The YOLOv8 model was trained for 100 epochs on this dataset and achieved a mean Average Precision (mAP) of 0.720 at 50% IoU (mAP@50) and 0.506 across IoU thresholds (mAP@50–95) on the sidewalk construction validation dataset. For inference, the confidence threshold was set to 0.250 and the IoU threshold to 0.700. Class-specific performance metrics revealed precision and recall values of 0.683 and 0.604, respectively, for horizontal scaffolding, and 0.789 and 0.717, respectively, for scaffolding poles. These results highlight the model’s robust detection capabilities, even in complex scaffolding setups.

### Construction-related objects detection

We propose to use an open-vocabulary object detection model to handle the variability of objects found at construction sites. Open-vocabulary object detection enables the recognition of a broad array of objects without the necessity for extensive training on specific categories [21]. The model used for this project is YOLO-World. YOLO-World is an advanced object detection framework based on the YOLO series, designed for real-time open-vocabulary object detection [21]. It integrates vision-language modelling to overcome the traditional YOLO limitation of detecting only predefined categories. The reason for selecting YOLO-World is due to the balance between speed and accuracy. It reaches high efficiency with 52.0 FPS on NVIDIA V100 GPUs and strong zero-shot detection performance with 35.4 average precision on the LVIS dataset [21]. Furthermore, once fine-tuned on a predefined set of construction-related categories, YOLO-World can be exported as a standard YOLO model for efficient, real-time inference on edge devices such as the NVIDIA Jetson board, enabling deployment without the computational constraints typically associated with vision-language models.

To tailor YOLO-World for construction detection, we curated a specialised vocabulary focused on common construction objects. Notably, we included multiple descriptors for the same object to account for variations in appearance due to lighting conditions and the model’s sensitivity to colour nuances. For instance, terms like “green wall”, “dark green wall”, and “green construction wall” were used to describe construction site wall dividers that may appear differently under varying illumination. In an open-vocabulary setting, any single textual description might not perfectly align with the learned visual features [22]. Supplying multiple descriptions increases the likelihood that at least one textual embedding will closely match the object’s visual representation.

Objects representing construction sites include traffic cones, traffic barricades, traffic barrels, and construction wall dividers. To detect these objects, the terminology used for the construction site objects were: “traffic cone”, “orange and white striped traffic barrier”, “construction barricade”, “traffic barrier”, “white traffic barrier”, “red traffic barrier”, “orange traffic barrier”, “red traffic barricade”, “white traffic barricade”, “red and white barricade”, “green construction wall”, “construction wall”, “green wall”, “dark green wall”.

A prominent issue in open-vocabulary object detection is overgeneralisation, which occurs when a model extends its decision boundary too broadly, inadvertently including areas of the feature space that do not correspond to any recognised class [23]. This issue can lead to incorrect classifications where the model mistakenly labels objects with similar features as belonging to the same class. For example, in the case of detecting a “white traffic barricade”, if a white car is the only white object present, the model may erroneously classify the car as the barricade due to the overly generalised decision boundary.

To mitigate the problem of overgeneralisation, we introduced null classes. A null class is defined as a category that the model is not specifically interested in detecting, but which can help improve the performance for classes of interest [24]. Incorporating these additional classes refines the model’s decision boundaries, improving its ability to distinguish visually similar objects. Our null classes were identified through testing with various images and common misclassifications. For example, objects such as “fire hydrant” which was frequently misclassified as “traffic cone” as well as “grassland” which was often misclassified as a “green construction wall” were included as null classes to enhance model differentiation capabilities.

The following null classes were integrated into the model based on empirical testing to address common misclassifications: “car”, “white car”, “truck”, “bench”, “fire hydrant”, “computer monitor”, “tree”, “tree canopy”, “building”, “grass”, and “grassland”.

Moreover, customised confidence scores tailored to specific terms were used to improve detection accuracy in object detection models. A key characteristic of YOLO-World is its higher confidence levels for terms corresponding to classes in the training dataset [25]. To ensure accurate predictions for each term, adjusting confidence thresholds at the term level is important.

To manage variability in confidence scores, we established three distinct categories through internal testing. Each category addresses different classes of objects, allowing the model to adapt its detection thresholds based on specific requirements. A threshold range of 0.005–1.0 was applied to the following classes: “green construction wall”, “dark green wall”, and “construction wall”. These objects are challenging to detect due to their subtle textures and colours that often blend into the environment. Lowering the threshold allows the model to pick up more instances of these objects, ensuring they are not overlooked during detection. A threshold range of 0.03–1.0 was used for the following classes: “construction barricade”, “red traffic barricade”, “white traffic barricade”, “car”, “bench”, “tree”, and “building”. These objects typically fall within the expected confidence range of YOLO-World’s scoring system, where confidence scores often stay under 0.1 [25]. This threshold ensures these objects with moderate detection confidence are captured effectively without compromising precision. A threshold range of 0.12–1.0 was used for the following classes: “red traffic barrier”, “orange traffic barrier”, and “traffic cone”. These objects are more likely to generate false positives due to visual similarities with unrelated objects like fire hydrants, trash bins, or red tiles on sidewalks. By setting a relatively high confidence threshold, the model filters out low-confidence predictions, reducing false positives and improving overall detection accuracy. Overall, by strategically defining and integrating redundant classes based on common misclassifications and customised thresholds for different classes, the decision boundaries of a model will be enhanced, and confusion among similar objects will be reduced, ultimately leading to better performance in real-world applications.

### Construction sign detection

Text is an integral part of urban environments, commonly found on street signs, advertisements, murals, and shop names. For pBLV, interpreting these texts presents unique challenges. Construction sites, in particular, often include critical signage that conveys important safety information, such as warnings, directions, or restricted access notices. To assist pBLV in interpreting construction signs, we employ OCR technology.

We selected PaddleOCR 3.0 [26] for this project, as it is one of the most reliable OCR tools available. Numerous studies have demonstrated its effectiveness in various applications, including licence plate recognition [27], health code recognition [28], and the detection of urban scene texts [29]. PaddleOCR’s robust performance makes it an ideal candidate for identifying construction-related text in dynamic and challenging urban environments. Despite its strong text detection, not all text detected in construction environments is relevant. For instance, advertisements, graffiti, or unrelated street signs can appear in the OCR output, creating noise that complicates the identification of critical construction-related information. To address this, we implemented a filtering mechanism, proposed previously [30], to refine the PaddleOCR output to focus on relevant signs. To effectively filter relevant content, we developed a comprehensive dictionary of possible construction signs. This dictionary serves as a reference to verify the relevance of the text detected by PaddleOCR. Six student volunteers contributed to this effort by surveying ~50 construction environments, either in person or using Google Maps StreetView. Through these surveys, 171 construction signs were observed, and 64 unique strings relevant to construction were extracted. Examples of these strings include “Authorized Personnel Only”, “Caution: Construction Zone”, and “Road Work Ahead”.

The string similarity was calculated to compare the OCR detected text with the entries in the construction sign dictionary. This method leverages the Sorensen-Dice coefficient [31], a metric that effectively measures string similarity by comparing shared character sequences. For every text output by PaddleOCR, a similarity score was calculated against all entries in the dictionary. A threshold of 0.8 was set, meaning that any text with a similarity score exceeding this threshold was considered a match to a construction-related sign. This scoring mechanism ensures that minor variations in wording, caused by OCR inaccuracies, abbreviations, or changes in phrasing, do not prevent the accurate detection of relevant construction text. For example, even if a detected string reads “roadwrk Ahead”, the similarity score calculation would correctly identify it as matching the intended sign, “Road Work Ahead”.

### Experimental setup

We conducted experiments to evaluate the system’s effectiveness in outdoor environments. Four volunteers wore a smart wearable device designed for pBLV, the Visually Impaired Smart Service System for Spatial Intelligence and Navigation (VIS^4^ION), to collect video footage around construction sites from various angles. VIS^4^ION is a personal mobility solution offering a customisable, human-in-the-loop, sensing-to-feedback platform that provides real-time functional assistance [32–36]. The system is a wearable backpack equipped with multiple sensors, including two cameras mounted on the shoulder straps. The cameras are Arducam 1080P Low Light Ultra Wide-Angle USB Cameras [37], recording video at 30 fps with a resolution of 1920 × 1080. The cameras have a diagonal field of view (FOV) of 160°. VIS^4^ION utilises an NVIDIA Jetson Orin NX as its processing unit—a powerful embedded AI computer designed for generative AI, computer vision, and advanced robotics applications [38]. All collected video data are processed directly on this device. The experimental setup consisted of two parts: a static test and a dynamic test, both conducted in NYC to evaluate the system’s detection capabilities for construction site objects.

#### Static testing setup

**Figure 1.**
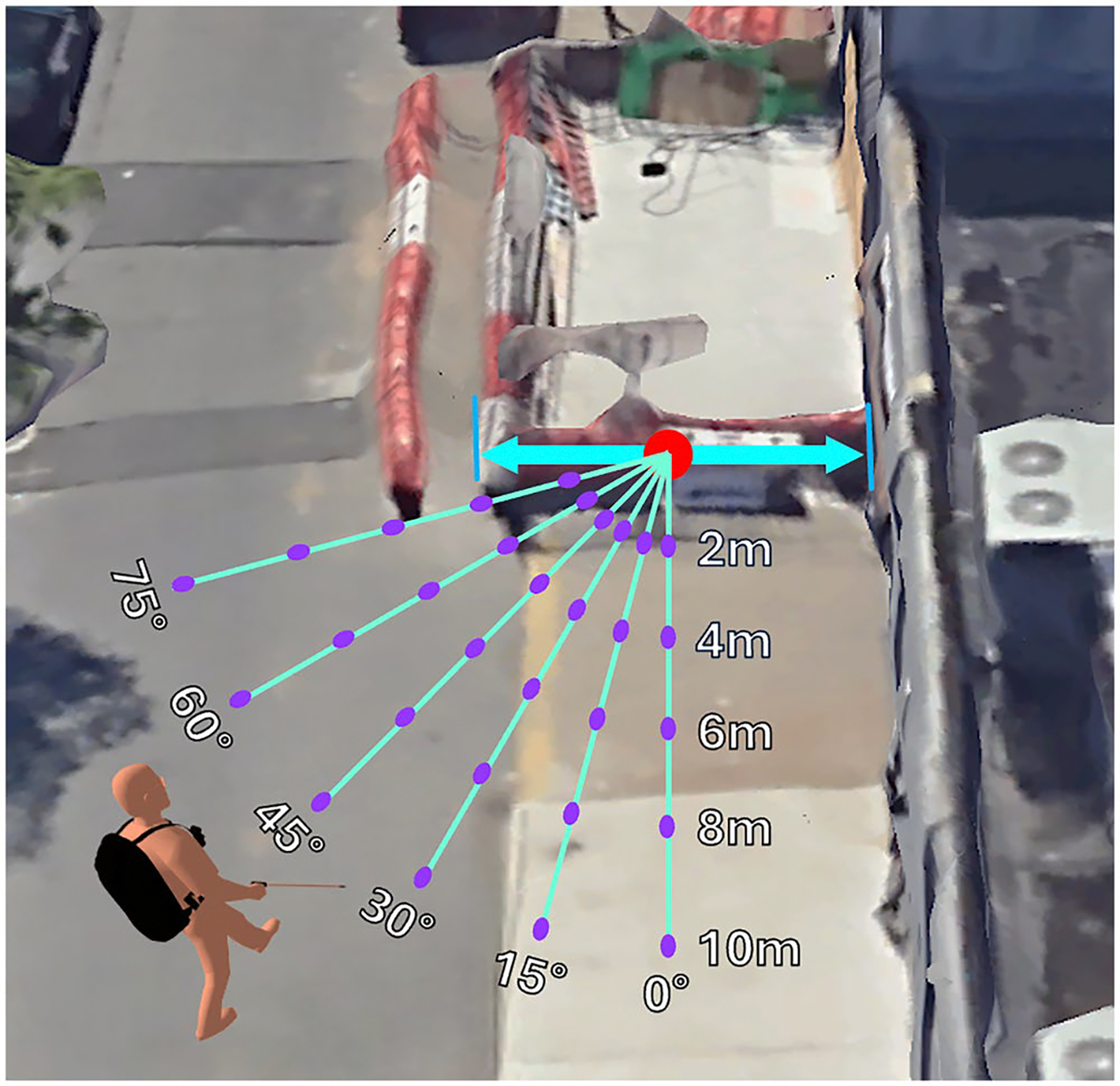
A graphical illustration of the data collection procedure at a construction site. The blue arrowed line represents the width of one end of the construction site, with the red point at its centre serving as the reference point. Six green lines indicate measurement lines from different angles, with their angles labelled in white text. Purple dots mark the locations where the volunteer pauses for 5 s. An orange human figure wearing the VIS4ION system is positioned near the 45° and 10 m mark. This figure is for conceptual illustration purposes; the human figure’s scale does not accurately reflect the actual scale of the environment.

In the static testing, four volunteers visited seven distinct construction sites across NYC streets, each characterised by specific construction elements. Sites 1 through 7 prominently featured sidewalk scaffolding, traffic barrels, barricades, traffic cones, construction site dividers/temporary walls, construction signs, and a combination of multiple construction elements, respectively. These sites represent the most commonly observed types of construction sites in NYC. At each location, volunteers collected videos from various angles and distances. The procedure for video collection is illustrated in Figure 1. At the construction site, the width of one end of the site was measured, as shown by the blue arrowed line in Figure 1. The centre of the blue arrowed line was designated as the reference point for angle and distance measurements.

A virtual line, perpendicular to the blue arrowed line and passing through the reference point, was defined as the 0° line, shown as the green line labelled 0° in Figure 1. The 15° line was defined as a line passing through the reference point, forming a 15° angle with the 0° line. Similarly, the 30, 45, 60, and 75° lines were defined in the same manner, as shown in the green lines with angle labels in Figure 1. Along each line, measurement points were marked at distances of 2, 4, 6, 8, and 10 m from the reference point. Some of these points are depicted as purple dots in Figure 1. Volunteers recorded first-person videos while walking along each virtual line and paused at every measurement point for at least 5 s to ensure stable capture. From each pause segment, a single key frame was extracted for evaluation to maintain consistency and reduce computational complexity. We selected the first frame that was stable during the pause interval, where motion blur and occlusion were minimal.

#### Dynamic testing setup

Dynamic testing is essential for evaluating the reliability and performance of systems in real-world urban walking scenarios. Unlike static testing that merely assesses the detection range using clear video frames, dynamic testing addresses additional ecologically valid complexities, such as motion blur, object occlusion, and varying lighting conditions, which influence the system’s robustness. Dynamic testing simulates real-world scenarios that systems will face daily, making it crucial for evaluating effectiveness and reliability.

In this testing, a volunteer wore the VIS_4_ION system and walked naturally along NYC streets. A volunteer completed a trip in the Downtown Brooklyn area, covering ~0.5 miles in about 9 min. The video data collection was conducted at the volunteer’s own comfortable walking pace. The recordings took place on a typical sunny summer afternoon in NYC, with average summer temperature, humidity, and sunlight conditions. No abnormal wind, noise, or shadow conditions were reported during the recording. This route encountered 8 different construction sites. Approximately one-fourth of this route required the volunteer to navigate areas impacted by construction sites. These included walking under sidewalk scaffolding, traversing narrower paths created by barricades, and rerouting due to road closures, etc. This route was set in a typical Downtown Brooklyn business area. Key features include encountering other pedestrians every few steps, illegally parked cars partially blocking the path, and trash piles scattered on the sidewalks. According to NYC Open Data [39], the sidewalk width along this route ranges from ~2.5 to 4.8 m.

All video frames were manually annotated. Frames are labelled as “non-construction site” if they do not contain a construction site on the user’s path within a reasonable detectable range. Construction sites located on the opposite side of the sidewalk are also labelled as non-construction site, as they do not affect the user’s immediate path. Similarly, the presence of isolated construction-related objects not actively used for construction purposes (e.g., traffic cones used to block parking) are excluded from labelling.

When a construction site appears on the sidewalk where the user is walking, frames are labelled as “construction site” from the moment the site becomes visually significant in the first-person video. We define visual significance as the point when at least one construction-related object occupies more than 3% of the image height or width. This threshold was determined empirically: the smallest detectable object in our construction-site detection model, a traffic cone, is clearly recognisable from about 10 m away, at which distance it occupies roughly 3% of the frame width. Larger objects, such as scaffolding or green walls, surpass this threshold from even greater distances. Therefore, applying the 3% threshold ensures that construction-site frames are labelled at distances of at least 10 m. Frames continue to be labelled as “construction site” until the video recorder has clearly passed the site and no relevant construction-related elements remain in view. This approach supports fairer assessment of detection performance and better reflects real-world conditions where early visual cues are essential for safe navigation.

To enhance prediction accuracy and temporal consistency, we applied a post-processing technique known as temporal majority voting, commonly used in video analysis to smooth frame-by-frame predictions [40]. In our case, the system outputs a binary prediction for each frame—either “construction site” or “non-construction site”. Temporal majority voting aggregates the predictions across a sliding window of K consecutive frames and assigns the most frequent label within that window as the final output for each target frame. We experimented with a range of K values from 1 to 100 to evaluate the impact of this technique on overall detection performance.

Moreover, to better approximate how users experience detections in real-world conditions, we also evaluated an event-based evaluation approach. This method treats detection outputs as discrete events rather than isolated frame-by-frame decisions, reducing the inflation of false positives caused by short-term noise.

An alarm event was defined as a sequence of at least 10 positive detection frames within a 30-frame (1-s) window. Consecutive alarm events were required to be at least 30 frames apart to be considered distinct. This reflects the perceptual reality that users interpret a stream of system outputs as meaningful events, not as instantaneous frame classifications. To calculate the event-based false alarm rate, we compared predicted construction site alarms to the ground truth. Alarms were considered true positives if they occurred in regions labelled as construction sites in the ground truth, otherwise they were marked as false alarms.

### Evaluation metrics for the static testing

In static testing, 30 unique frames were extracted at a construction site, each frame was extracted from a measurement point, representing a specific view based on angle and distance, such as 0° at 2 m, 0° at 10 m, 75° at 6 m, and so on. For each measurement, at least 5 s of video were recorded, generating multiple frames. The extracted frame was selected as the first frame where the camera stabilised, and the image became clear. These frames were used to evaluate the system’s performance in static conditions using three key metrics: accuracy, angular offset, and distance.

#### Accuracy

Accuracy measures the overall success rate of identifying construction-related objects or signs within a construction site. Since testing frames are extracted from measurement points, a construction site must exist within the frame. A successful detection is when the proposed model also classifies these frames as containing a construction site.

The accuracy across the tested range is calculated using the following formula:

$$\text{Accuracy}=\frac{\text{Number}\;\text{of}\;\text{Successful}\;\text{Detected}\;\text{frames}}{\text{Total}\;\text{Number}\;\text{of}\;\text{Frames}}\times 100\%$$

This metric serves as a crucial indicator of the model’s overall accuracy and reliability in object detection.

#### Angular offset

Angular offset is a critical metric used to evaluate a system’s ability to detect objects at varying angles. It reflects the effectiveness of the system in identifying construction-related objects or signs when observed from different angles.

$$\text{Angular}\;\text{offset}=\frac{\text{Number}\;\text{of}\;\text{Frames}\;\text{at}\;\text{a}\;\text{Given}\;\text{Angle}\;\text{with}\;\text{Successful}\;\text{Detections}}{\text{Total}\;\text{Number}\;\text{of}\;\text{Frames}\;\text{at}\;\text{that}\;\text{Angle}}\times 100\%$$

This metric is determined by calculating the ratio of successful detections at a specific angle to the total number of frames tested at that angle. The definition of successful detection is the same as above, that the predominant construction object(s) in that frame is detected. At each construction site, angular offset was measured across five detection angles: 0, 15, 30, 45, 60, and 75°.

#### Distance

This metric is determined by calculating the ratio of successful detections at a specific distance to the total number of frames tested at that distance. The definition of successful detection remains the same, requiring that the predominant construction object(s) in a frame be detected. At each construction site, distance was measured across five distances: 2, 4, 6, 8, and 10 m. This metric is crucial for understanding how detection performance correlates with distance, helping to evaluate how varying ranges impact the system’s ability to detect objects.

### Evaluation metrics for the dynamic test

In the dynamic test scenario, we relied on several performance metrics to gauge the model’s ability to classify each video frame accurately. These metrics are defined based on how well the system distinguishes between “construction site” and “not construction site” frames.

- **Accuracy**: A fundamental measure of the proportion of frames correctly classified. It reflects the overall reliability of the system in identifying construction-related frames while avoiding misclassifications of non-construction frames.
- **Error rate**: Determined by the proportion of frames that are misclassified, providing an inverse perspective on the model’s performance. A higher error rate signals a greater number of incorrect classifications.
- **Specificity**: Illustrates how effectively the model identifies non-construction frames. It reflects the model’s accuracy in filtering out frames that do not contain construction activity, ensuring fewer false positives.
- **Precision**: Focuses on frames predicted as “construction site”, indicating how many of these predictions are actually correct. It provides insight into the model’s ability to minimise false alarms.
- **Recall**: Measures the fraction of true construction-site frames that the model successfully identifies. This metric highlights the model’s capability to detect all relevant instances of construction activity.
- **F1 score**: Represents the harmonic mean of Precision and Recall, offering a single measure that balances these two aspects of performance. It is particularly useful when seeking to optimise both the identification and correctness of “construction site” predictions.

## Results

### Static testing

The overall accuracy across all measurement points and all seven construction sites includes 210 measurement points in total and results in 186 correct detections (88.56%). The breakdown of accuracy at each measurement point is shown in Table 1.

The angular offset was evaluated at incremental angles from 0 to 75° across the seven sites. The mean success rates (±*SD*) were as follows: 0°, 91.4% (±7.0%); 15°, 94.3% (±7.0%); 30°, 91.4% (±17.1%); 45°, 88.6% (±16.7%); 60°, 88.6% (±16.7%); and 75°, 77.1% (±23.2%). These results demonstrate that the detection accuracy is highest within the range of 0–30°, consistently above 90%. However, as the angle increases beyond 30°, the accuracy shows a gradual decline, with a notable drop to 77.1% at 75°. The higher accuracy at smaller angular offsets suggests that the system performs best when the construction site is viewed in a more perpendicular manner.

The distance was evaluated in increments of 2 m, from 2 to 10 m, yielding mean success rates (±*SD*) of 100% (±0%) at 2 m, 100% (±0%) at 4 m, 95.2% (±6.7%) at 6 m, 83.3% (±12.8%) at 8 m, and 64.3% (±16.0%) at 10 m. Overall, these results demonstrate a consistent decrease in detection coverage with increasing distance, suggesting that the model’s performance is robust at closer ranges but becomes progressively challenged as the target objects recede from the camera.

**Figure 2.**
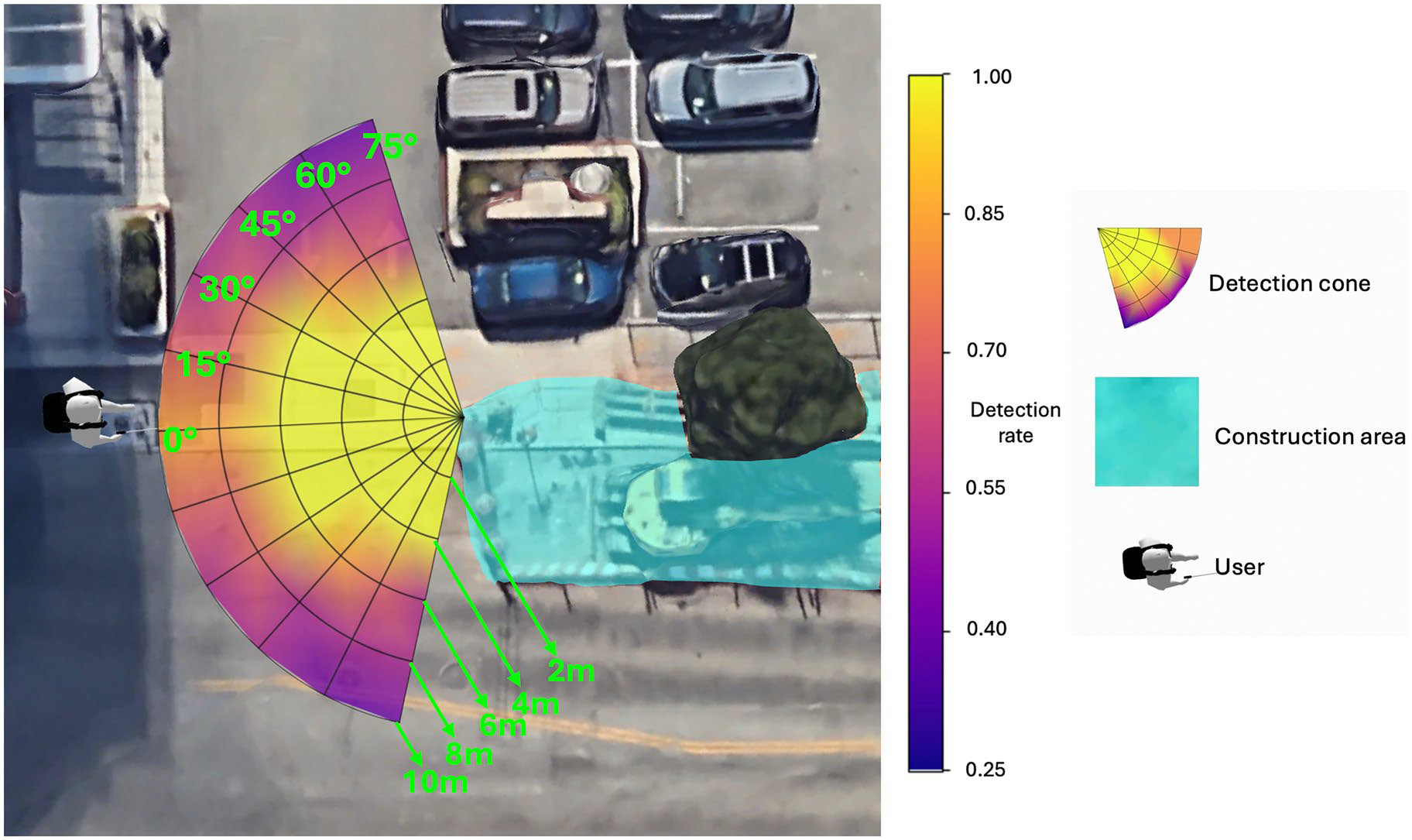
Visualisation of the system’s construction site detection range. The coloured fan-shapes depict detection success rates at different angles and distances: yellowish areas indicate higher success rates, while purplish areas show lower rates. The green coloured area represents the construction area.

This detection range is illustrated in Figure 2, where a colour map visualises the detection success rate at various locations. A yellowish colour indicates a high accuracy, while a purplish colour represents areas with a lower accuracy. From Figure 2, it is evident that accuracy is higher when the objects are close to the user or directly in front of them.

Detection performance varies based on the predominant features of the construction site. For construction sites characterised by wall dividers, traffic barrels, or traffic cones, the system successfully detected objects across all measurement points. The accuracy at these sites was 100% (±0%). The angular offset reached 100% across the entire tested range, from 0° to the maximum testing distance of 10 m, where the accuracy remained 100%.

For construction sites with predominant features of sidewalk scaffolding, detection failures occurred only at 10 and 8 m for the 75° angle. This indicates that 75° is a particularly challenging angle for the model to detect objects correctly at greater distances.

For construction sites dominated by traffic barricades, unsuccessful detections were observed at 10 m for the 30, 45, 60, and 75° angles, at 8 m for the 45 and 75° angles, and at 6 m for the 75° angle. Overall, this type of construction site proves more challenging to detect at a distance of 10 m or at the 75° angle.

For OCR-based reading, successful text recognition occurred only within 4 m. Within this range, detection was consistently successful across all angles.

### Dynamic testing

We collected a test video containing 14,867 frames, of which 4038 were labelled as construction-site frames and 10,829 were labelled as non-construction-site frames.

Each frame was processed through the decision-making framework to determine whether it was correctly classified as a construction site. The results showed 87.26% accuracy, 74.50% precision, 80.73% recall, and 77.49% F1 score. Results are shown in Table 2.

### Temporal majority voting

We evaluated the effect of varying the majority voting window size *K* on detection performance. Table 3 reports accuracy, precision, recall, and F1 score for *K* values from 1 to 100 frames. The largest relative F1 improvements occur when *K* increases from 1 to 5 (+5.0%) and from 5 to 10 (+1.2%). At K = 30, the F1 score is +1.4% higher than at K = 20. At K = 50, the gain is +0.4% compared with K = 40. From K = 90 to K = 100, the curve is almost flat, with values changing by only about 0.001 and oscillating slightly. The highest F1 score observed is 0.876 at K = 90. While the maximum F1 score is achieved at K = 90, the improvement over K = 50 (0.855) is only +2.1%, despite nearly doubling the decision latency.

#### Ablation study

To assess the individual contributions of each detection module, we conducted an ablation study using the dynamic walking video dataset. For each configuration—removing one or more modules from the full system—we measured the detection rate (recall), false alarm rate, and the estimated false alarms per minute. The modules evaluated include the open-vocabulary YOLO-World detector, the scaffolding-specific YOLO model, and the OCR-based construction sign recognition module. Results are summarised in Table 4.

The results show that the full system achieves the highest detection rate, highlighting the importance of integrating all three modules. Removing the YOLO-World or scaffolding YOLO modules leads to substantial drops in detection performance. The OCR module alone provides minimal coverage, as most construction sites include physical features in addition to signage. Any single module in isolation results in lower detection and/or increased false alarms, underscoring the necessity of a combined approach for robust construction site detection in real-world scenarios.

#### Event-based accuracy

Across the entire walking video, we identified a total of 28 discrete alarm events. Of these, 14 were true positives—instances where the system correctly detected an on-path construction site. The remaining 14 were false alarms. Upon further breakdown, 8 of these false alarms were due to construction sites on the opposite side of the street. Two alarms were triggered by the presence of construction-related objects, such as cones or barriers being used for non-construction purposes. The remaining 4 were complete false positives, where the system incorrectly recognised enough visually similar elements.

We also categorised the false alarms by the system module responsible. Of the 14 false alarms, 7 were caused solely by the YOLO-World module, 4 by the Scaffolding YOLO detector, and 3 resulted from false positives from combined misclassifications.

The total alarm rate is 2.8 events per minute. The false alarm rate is 1.4 events per minute. However, if we exclude technically correct detections that were not labelled due to the conservative annotation protocol, only 4 events remain as complete false positives—yielding a more realistic false alarm rate of 0.4 events per minute. This demonstrates that the system performs robustly in dynamic conditions when event-based metrics are used. The result is summarised in Table 5.

## Discussion

Static testing confirmed that the proposed method can reliably detect construction sites under a wide range of viewing conditions, with performance highest at closer distances and near-frontal approach angles. Large, high-contrast elements such as construction dividers, traffic cones, and barrels were recognised most consistently, while smaller or visually complex objects like barricades and signs were less reliable—particularly at oblique angles or longer ranges. These trends underscore the role of object size, contrast, and visibility in determining detection success.

In dynamic testing, performance improved with temporal majority voting. A 50-frame window provided the best balance between predictive accuracy, responsiveness, and computational efficiency, as detailed in Section Benefits of temporal majority voting.

Construction-site detection performance varied with the user’s approach angle. At a 0° angle (perpendicular to the construction site), every site was correctly identified out to 10 m—except those dominated by signage, whose text is too small at that distance for OCR to read reliably. Large, high-contrast elements such as green construction walls were detected consistently even at greater distances and oblique angles. Detection of traffic cones and barrels was likewise highly accurate because YOLO-World’s training dataset contains many examples of these, boosting confidence and precision [41]. By contrast, objects like traffic barricades posed greater challenges, as they are likely under-represented in their training dataset, particularly when viewed obliquely.

In the ablation study, the OCR module did not significantly increase overall accuracy, as construction signs are often colocated with other detectable construction objects, which are identified first. This explains the minimal change in detection rate when OCR is removed. However, OCR remains important because a sign explicitly stating “construction site ahead” provides an unambiguous confirmation, especially in visually ambiguous scenes. While not all sites have signage, when present, it can both confirm detection and potentially reduce false positives, making OCR valuable despite its modest quantitative impact.

Moreover, one key challenge observed was false positives in open-vocabulary object detection due to overgeneralisation. For instance, the model frequently misclassified objects with similar visual features, such as mistaking streetlight poles or silver window frames for scaffolding poles. To mitigate this, we developed a specialised YOLO model focused on scaffolding pole detection, which substantially reduced false positives and improved precision.

In the next sections, we further analyse the false alarm rate, causes of detection inaccuracies, including specific challenges related to object orientation, occlusions, and model confidence thresholds.

### False positive analysis

A key metric in evaluating assistive detection systems is the false alarm rate, which reflects how often the system mistakenly signals the presence of a construction site. In our analysis, we identified three primary sources contributing to the false alarm rate.

First, our initial analysis used a frame-based approach, where any individual frame that falsely predicted a construction site was counted as a false alarm. However, this method can overestimate the impact of false alarms on the user experience. Since the system processes video at 30 frames per second, even a brief misclassification lasting half a second can lead to 15 falsely labelled frames. In practice, users perceive alerts as discrete, momentary events—not as a continuous stream of frame-level decisions. Therefore, repeated false positives across adjacent frames are often interpreted by users as a single false alarm. Frame-by-frame detection also introduces inconsistencies due to environmental noise, motion blur, or partial occlusions. A single frame may be misclassified, only to have surrounding frames correctly identify the scene. These transient errors can be smoothed using temporal filtering techniques, such as majority voting over a moving window. For example, our ablation study reported a relatively high frame-based false alarm rate, but that analysis did not incorporate any temporal smoothing. Applying a 50-frame majority voting window increased accuracy to 92%, and further improvements were observed using event-based detection.

**Figure 3.**
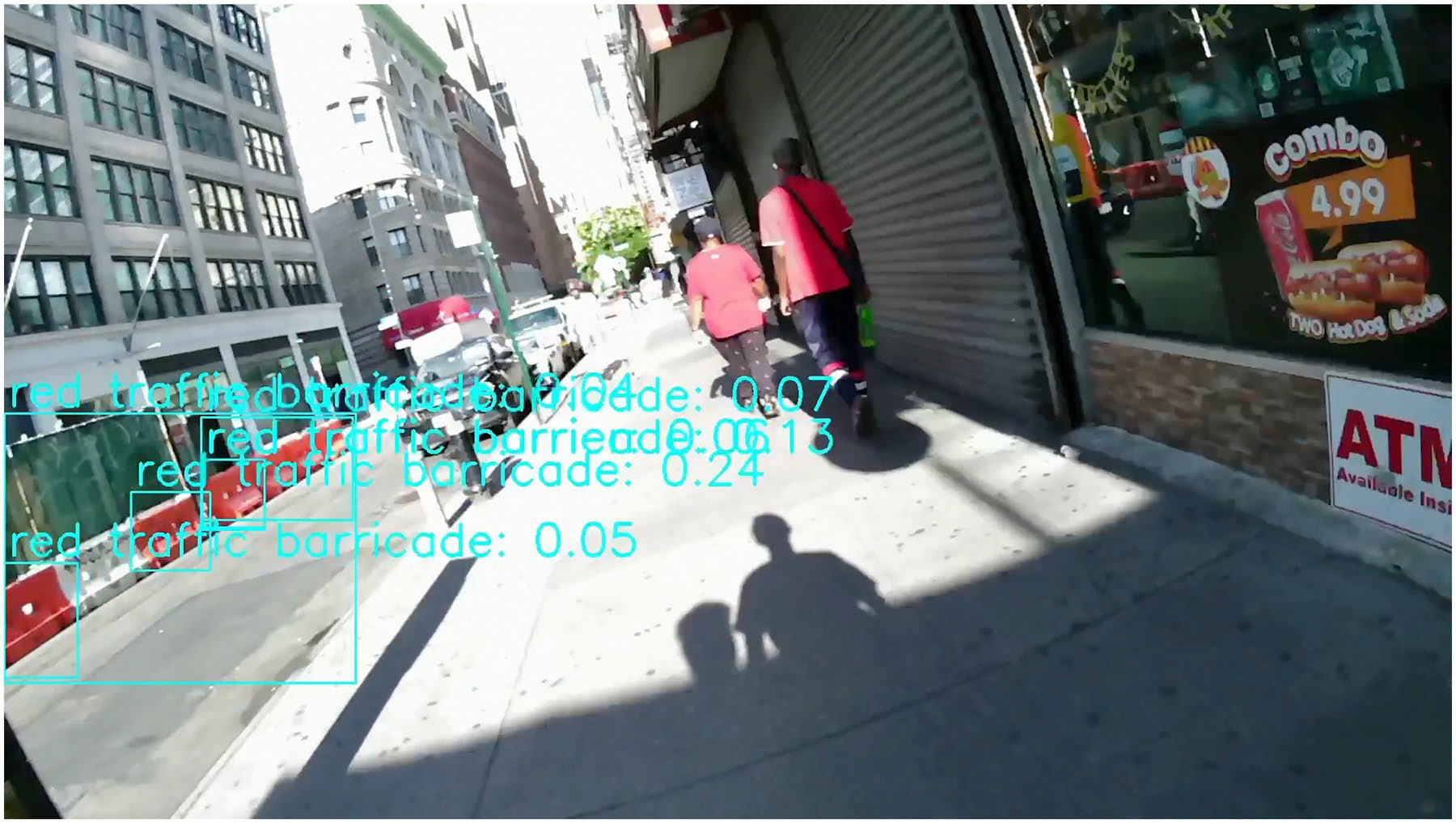
A frame showing a construction site on the opposite side of the sidewalk. While this is a genuine construction site and was technically detected correctly, it does not affect the user’s walking path, so the frame is labelled as no construction.

Second, construction sites located on the opposite side of the sidewalk or street were labelled as negative cases because they did not obstruct the user’s movement. However, these were still genuine construction zones that the system correctly identified. Figure 3 is an example of the construction site on the opposite side of the sidewalk. Because our evaluation focused only on obstacles directly in the user’s path, these valid detections were counted as false positives. This conservative labelling strategy emphasises practical relevance but can artificially inflate the false alarm rate. On the other hand, it also demonstrates the system’s ability to detect construction activity at longer distances, which may be useful for early warnings or broader situational awareness. Future work should explore how to balance extended detection range with alert precision.

**Figure 4.**
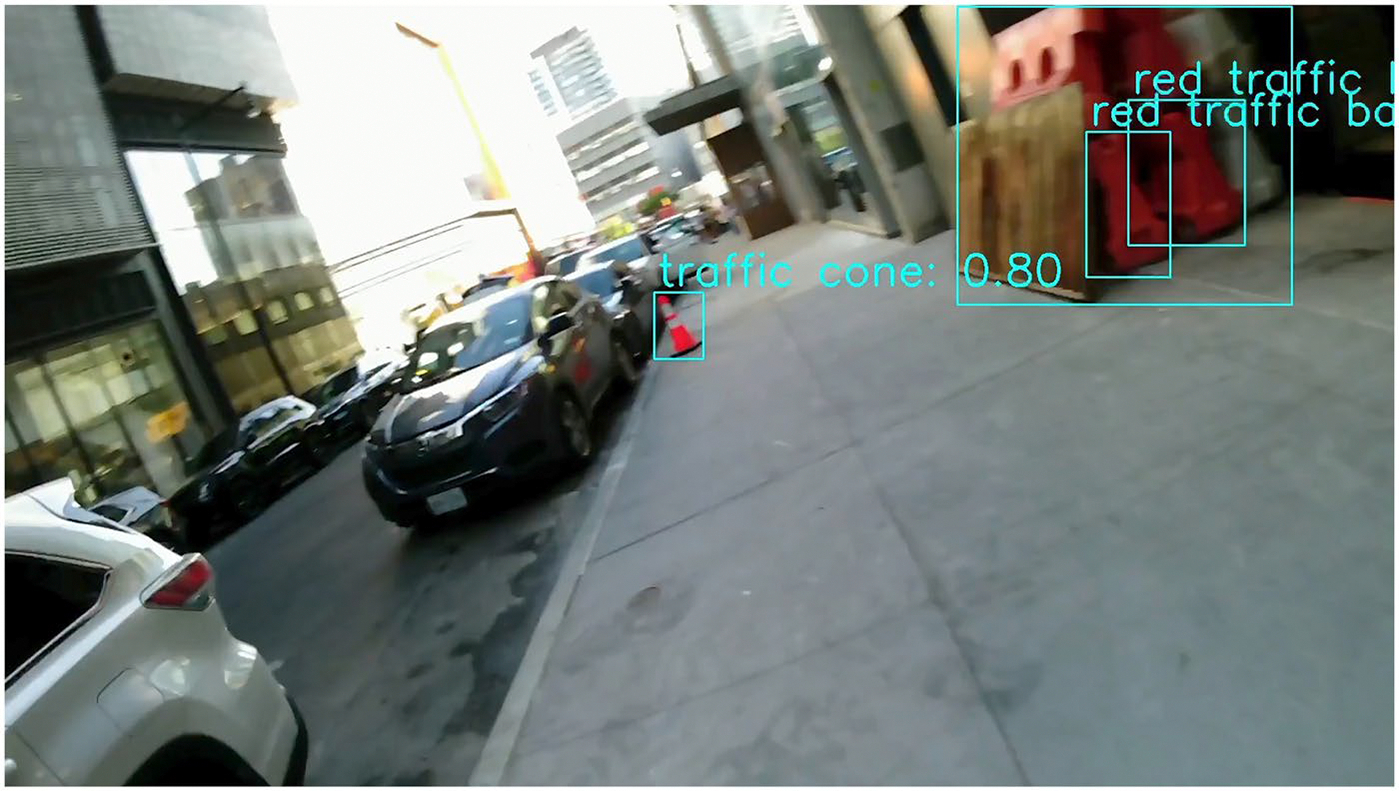
A frame showing construction-related objects successfully detected; however, these objects are not associated with active construction sites.

Third, as shown in Figure 4, construction-related objects such as cones, barriers, or materials may appear even in the absence of active construction. For example, traffic cones might be used to reserve parking spaces or redirect traffic temporarily. These instances are excluded from our ground truth labels, but the system may still detect them and trigger a false alarm. While this highlights the model’s high sensitivity to construction-related elements, it also reveals the challenge of distinguishing true construction zones from unrelated uses of similar objects. Future improvements may address this limitation through contextual reasoning or temporal consistency checks.

In total, 14 false alarm events were observed during the full test session. Of these, 8 were due to the detection of construction zones on the opposite side of the street, 2 were caused by inactive but present construction-related objects, and 4 were complete false positives. If we exclude the technically correct but ground-truth-excluded cases and count only true classification errors, the event-based false alarm rate drops to ~0.4 events per minute. In summary, most false alarms are due to evaluation strategy or environmental ambiguity rather than model failure.

### False negative analysis

#### Motion blur

**Figure 5.**
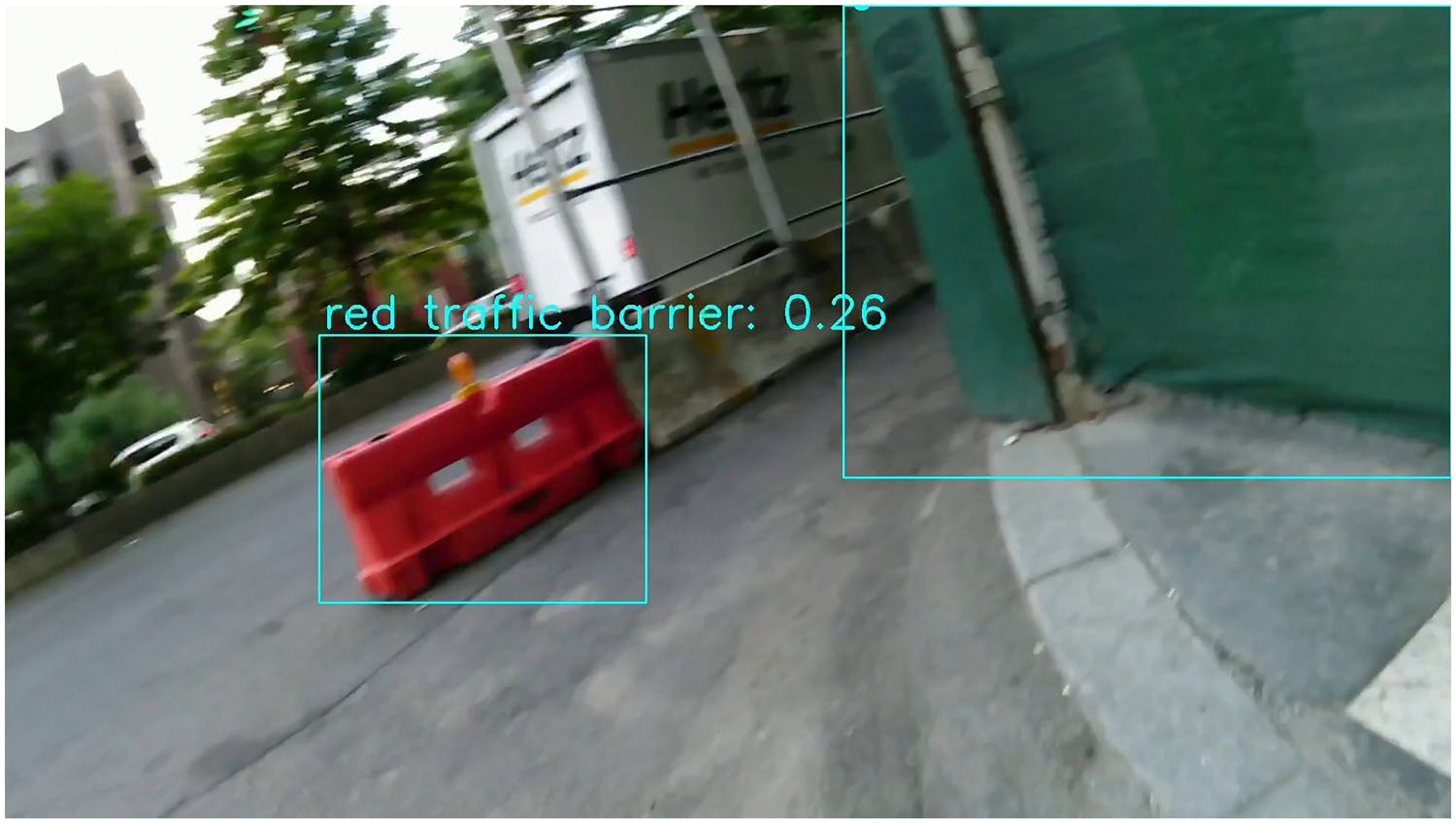
A frame showing the effect of motion blur on object detection. In blurred visual images, while some objects can still be detected such as the red traffic barrier and the green wall, others are not, including several instances of scaffolding poles.

Motion blur can sometimes cause problems with detection. Even though a frame rate of 30 frames per second works well for most walking scenarios, it struggles when the user makes sharp turns. During a turn, the angular velocity becomes high, and this creates motion blur in the video. As shown in Figure 5, motion blur during a turn can make it difficult to detect scaffolding poles. For example, in the figure, the closest scaffolding pole is only about 4 m away, yet it is not detected. This is in contrast to static testing, where the pole at the same distance is successfully detected. Once the user finishes the turn and resumes walking in a straight line, the detection rate returns to normal. In Figure 5, only a traffic barricade and a green construction divider were detected, which is insufficient to meet the threshold required to be considered a positive detection.

To address this challenge, potential solutions could include integrating an Inertial Measurement Unit (IMU) to detect turns and temporarily ignore frames captured during high angular velocity. Additionally, implementing a quality metric, such as Peak Signal-to-Noise Ratio (PSNR) [42], which is a metric used to measure the quality of an image or video by comparing it to a reference version, could ensure that only high-quality frames are processed, reducing the likelihood of false negatives caused by motion blur. Another possible solution is a hardware upgrade to use a high-frame-rate camera. Capturing more frames per second improves motion accuracy, resulting in smoother visuals and less blurring of fast-moving objects. These combined measures should enhance the robustness and reliability of the detection system under dynamic conditions.

#### Unexpected change in construction objects appearances

**Figure 6.**
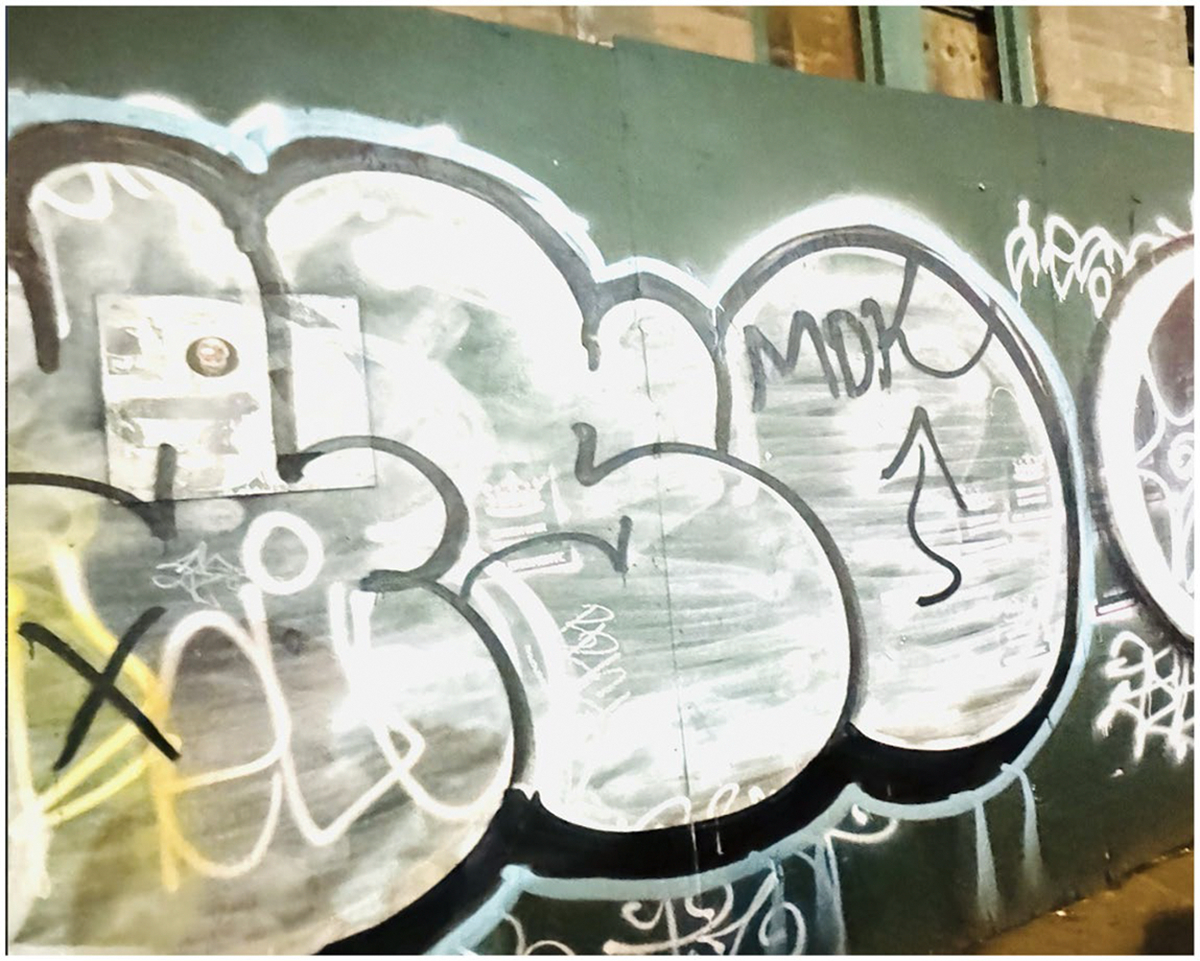
An example of a false negative from a green wall covered by graffiti.

Another cause of false negatives is the unexpected alteration in the appearance of construction objects. For instance, as shown in Figure 6, a construction wall divider became completely covered in colourful graffiti. While graffiti is technically illegal [43,44], it is widespread in urban environments, transforming the appearance of these walls. This transformation replaces the original uniformity of the walls with irregular and complex patterns, obscuring the key visual features that detection systems rely on for accurate recognition. Consequently, these visual alterations often prevent the system from correctly identifying construction sites, leading to false negatives.

To address these challenges, a strategy is to improve open-vocabulary object detection models by enriching the diversity and specificity of their prompt descriptions. Carefully crafting and fine-tuning prompts can help the system better recognise construction objects even when their appearances are altered by graffiti or other unexpected changes. Nevertheless, expanding the prompt set can sometimes lead to higher false positive rates, making it essential to systematically evaluate and optimise both prompt wording and decision thresholds for reliable performance. Compared to closed-set object detection models such as YOLO—which require extensive data collection and manual labelling to accommodate new appearances—open-vocabulary approaches are more adaptable and easier to update for evolving urban environments. However, closed-set models can still serve as a valuable fallback, especially in scenarios where open-vocabulary methods struggle. For instance, distinguishing between light poles and scaffolding poles remains difficult for open-vocabulary systems but can be handled reliably by a well-trained YOLO model.

Additionally, integrating texture and shape analysis into the detection algorithm can help address this challenge. Rather than relying solely on colour or patterns, the algorithm can focus on structural features such as the rectangular shape and dimensions of construction walls. This would enable the system to identify construction objects based on their inherent geometry, even when their surface appearance is altered.

#### OCR error

**Figure 7.**
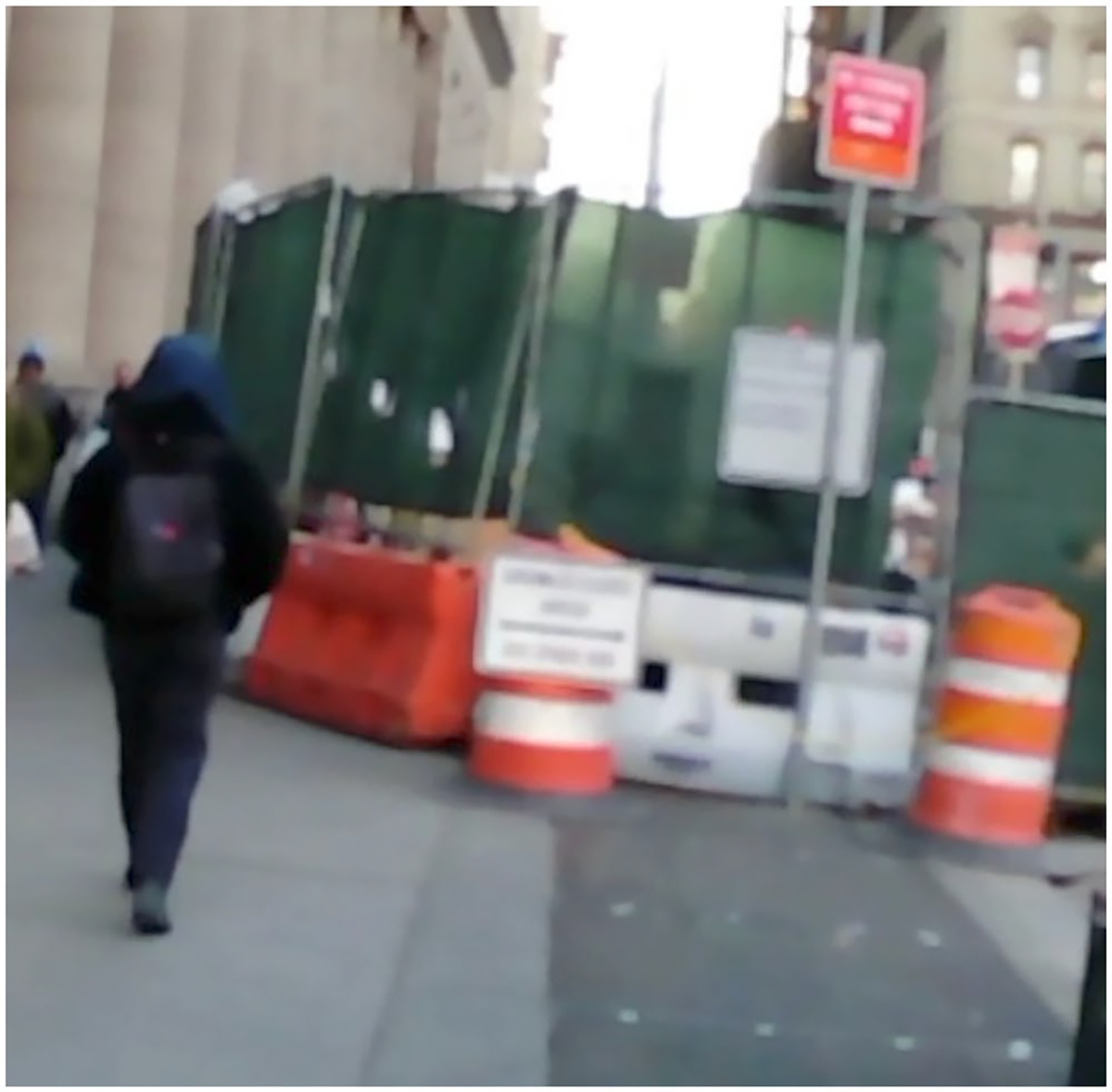
A cropped photo of a construction sign taken from 10 m away.

Construction sign detection can also result in false negatives, often because the text is unrecognisable to the OCR model. As shown in Figure 7, which depicts a cropped frame taken from 0° and 10 m away, the text on the sign is not legible—even to the naked eye. In this frame, a construction sign with black font on a white background is attached to a traffic barrel. The sign contains four rows of text: the first reads “Sidewalk Closed”, the second “Ahead”, the third features an arrow pointing to the left, and the fourth reads “Use Other Side”. However, due to the small font size and the blurriness of the image, the text cannot be recognised. As a result, the OCR model is unable to read the construction sign from any angle when the distance exceeds 8 m. At 6 m, only half of the angles result in successful detection, while within 4 m, the OCR model can successfully recognise the sign from all angles.

The primary cause of this blurriness is the camera’s lack of autofocus. In our setup, the camera only supports manual focal length adjustment, requiring the user to physically twist the lens to change focus. When the focal length is set too short, distant signs appear out of focus. In contrast, modern smartphones use advanced autofocus systems that automatically adjust the focal distance—typically at or near infinity for street scenes—so that distant objects, including construction signs, remain sharp and clear. As the user approaches a sign, the autofocus system dynamically refocuses as needed, keeping the text legible at any distance. Incorporating such an autofocus system would likely resolve most of the focus-related recognition problems observed in our experiments.

Moreover, another potential improvement is to implement a dedicated construction sign detector using an open-vocabulary detection model. This model can first identify and localise the region of the image that contains the construction sign. Once the sign is detected, the system can use this information to guide an autofocus-enabled camera to adjust its focal distance towards the sign. By ensuring that the sign region is in sharp focus, OCR performance can be improved—especially at medium to long distances where blur would otherwise render the text unreadable. This approach leverages physical lens adjustment to enhance clarity in the area of interest, enabling more reliable recognition even when the overall frame quality is suboptimal.

Finally, it may also be beneficial to explore more advanced OCR engines, such as Google Cloud Vision’s OCR [45], as commercial OCR solutions often provide better accuracy when compared to open-source alternatives.

### Implications for cognitive load and usability

The cognitive load theory posits that an individual’s capacity for information processing is limited, particularly when navigating complex or unfamiliar environments [46]. For pBLV, traversing urban spaces already imposes a substantial cognitive burden. These users must simultaneously coordinate multiple sensory modalities—such as auditory input, tactile feedback from a white cane, and proprioceptive awareness—while recalling route details and interpreting dynamic environmental cues. This multitasking can strain attentional resources, increase error rates, and elevate the risk of unsafe decisions, particularly when encountering unexpected hazards like construction sites. The proposed system is designed to reduce extraneous cognitive load by providing a single, high-level binary alert (e.g., “construction site ahead”), rather than requiring users to visually or tactually interpret environmental information. By converting complex visual scenes into simple, actionable signals, the system acts as a form of cognitive offloading, allowing users to focus on higher-level decision-making—such as rerouting or pausing for assistance—rather than hazard identification. This form of decision simplification is a critical design principle in assistive technology, as it supports user autonomy while minimising mental fatigue.

This cognitive framing also underscores the importance of alert timing and relevance. Alerts that are delivered too early, too frequently, or in response to irrelevant stimuli can increase rather than alleviate cognitive burden. By detecting construction zones at a meaningful range and filtering out non-actionable visual elements, the system improves both usability and trustworthiness. Although formal user studies are needed to validate these benefits empirically, the current system architecture is explicitly designed to offer concise, context-relevant feedback that enhances situational awareness and aims to support safer, more confident navigation for pBLV.

### Detection range

Our system currently detects construction sites with reasonable accuracy up to 10 m. Whether this detection range is optimal, however, requires further investigation and may depend on the user’s walking speed and reaction time. If the detection range is too short, users may not have sufficient time to receive and respond to a warning, increasing safety risks. On the other hand, if the detection range is too long, the system may detect construction sites or obstacles that are far ahead or not directly impacting the user’s route, leading to unnecessary alerts and potential alarm fatigue.

The choice of camera field of view influences these trade-offs. The VIS_4_ION system uses an ultra-wide-angle lens, which captures a broad area around the user. This wide perspective is helpful for identifying hazards approaching from the side or peripheral areas, enhancing situational awareness. However, ultra-wide lenses also make distant objects appear much smaller, which reduces the number of pixels available for detection. As a result, the accuracy for identifying small or faraway hazards will decline, as previously reported in the literature [47].

Moreover, a broader field of view can introduce additional challenges with false positives. For example, the system incorrectly identifies construction sites located on the opposite side of the street or sidewalk—sites that do not actually pose a risk to the user’s current path. Such misclassifications can decrease the reliability of the system and erode user trust.

Conversely, narrowing the field of view through video cropping may help reduce false positives by focusing more on what lies directly ahead of the user. This approach limits the inclusion of peripheral or background construction sites that do not affect the user’s path. A narrower view can also enhance effective detection range for forward-facing hazards, as objects appear larger and more prominent.

Additionally, the cropping strategy can be dynamic: when the user is walking straight, the focus area remains centred ahead; when the user initiates a turn, the focus can shift towards the turning direction to maintain relevant situational awareness. However, the trade-off is a potential loss of awareness for hazards that approach from the sides, which may be missed entirely. Optimising both the detection range and the camera’s field of view is crucial for balancing timely warnings with system reliability. Future work should systematically evaluate different camera configurations and detection thresholds to maximise real-world usability and minimise irrelevant or missed alerts.

### Ethical considerations

This study involved the collection of video data from public urban environments to evaluate a construction site detection system for assistive navigation. Although the dataset does not contain personally identifiable metadata, pedestrians may appear incidentally in the background of the videos. To address privacy concerns, we will apply automatic data anonymisation methods before any public release of the dataset. This includes techniques such as blurring facial features and licence plates, which ensure compliance with privacy standards while preserving the utility of the data for computer vision training and evaluation.

Importantly, our recent work has shown that such anonymisation has minimal impact on performance in tasks focused on environmental understanding rather than identity recognition [48]. Given that our system relies on the detection of environmental features rather than people, it is reasonable to expect that anonymisation will not significantly affect construction site detection performance.

### Open-vocabulary detection versus vision-language models

#### Value of on-device systems

Our findings highlight that YOLO-World, as an open-vocabulary detector, can be deployed directly on-device, providing fast and reliable inference without dependence on cloud servers. In contrast, many vision–language models are typically deployed using cloud-based infrastructure that relies on powerful GPUs, which may introduce latency and dependence on stable internet connectivity. While recent research has explored real-time video understanding with large models [49,50], these systems are generally evaluated using server-grade hardware, and their performance characteristics on lightweight wearable devices remain less well studied.

On-device deployment ensures that the system remains functional even in areas with weak or no connectivity, while also preserving privacy by keeping video data local to the device. This distinction is relevant for people with blindness or low vision, for whom connectivity disruptions may affect the reliability of assistive feedback during travel. In addition, on-device approaches are more scalable: once deployed, each additional user can operate independently without consuming centralised resources. This reduces costs for end users and manufacturers, making on-device deployment a practical and sustainable pathway for large-scale adoption.

#### Limitations compared to vision–language models

At the same time, it is important to acknowledge that open-vocabulary detectors have limitations relative to vision–language models. VLMs are trained on broad multimodal datasets and excel at flexible, language-driven reasoning tasks (e.g., answering open-ended questions or integrating visual context with prior knowledge). They also demonstrate higher semantic generalisation for rare or abstract categories, where open-vocabulary detectors may fail without targeted training or carefully curated prompts. In this sense, VLMs currently provide broader functionality and greater accuracy for general-purpose scene understanding, whereas open-vocabulary models are optimised primarily for object detection and spatial localisation.

#### Complementary roles for assistive technology

We view these two paradigms as complementary rather than mutually exclusive. Vision–language models can provide rich, descriptive scene interpretation when cloud connectivity and resources are available, while open-vocabulary detection ensures reliable, frame-by-frame monitoring on-device in safety-critical scenarios. The coexistence of both approaches may ultimately provide the most robust solution for people with blindness or low vision, combining the breadth of VLMs with the efficiency and real-time reliability of lightweight detectors. Our contribution lies in demonstrating that open-vocabulary detection is not only feasible on wearable hardware but also particularly well suited for continuous monitoring of environmental hazards, thereby filling a critical gap left by current VLM-based solutions.

### Limitations

All testing images were collected on sunny or cloudy days under typical daylight conditions. This setup limits the range of environmental factors that might impact the detection results, such as extreme lighting variations or the presence of rain. Moreover, performance under occlusion was not systematically evaluated. In real urban environments, target objects are often partially occluded by other pedestrians, vehicles, or street furniture. The extent to which such occlusions affect recognition failure remains unknown. Furthermore, the generalisability of the proposed approach requires additional testing across a broader range of environmental conditions and urban contexts.

While open-vocabulary object detection opens a new direction for construction site detection and this study demonstrates its promise as part of a larger assistive sensing pipeline, several limitations remain. One limitation, as discussed earlier, is overgeneralisation. Without mitigation strategies, detection accuracy can be substantially lower than that of a YOLO model trained on a well-labelled, task-specific dataset. Another limitation concerns viewpoint dependency. For barricades, detections are reliable at distances up to 10 m at approach angles of 0 and 15°, but the effective detection range is reduced to ~4 m at angles of 60 and 75°. In contrast, traffic barrels and traffic cones do not exhibit this behaviour, as their visual appearance remains largely invariant across viewing angles. The shift in visual characteristics between frontal and side views of barricades affects detection performance. For example, the frontal view typically appears as a flat rectangular panel, whereas the side view resembles an “A” shape, which complicates recognition at oblique angles. To mitigate false positives, we introduced redundant null classes that absorb common misclassifications, such as green construction walls being confused with grassland. However, identifying and maintaining such null classes is time-intensive and inevitably incomplete, leading to residual inaccuracies. These challenges highlight the need for more refined techniques to improve discrimination between visually similar objects and to enhance robustness in real-world deployment.

Finally, the scope of this study is limited to a single sensing platform and model configuration. All experiments were conducted using the VIS4ION backpack system equipped with USB cameras, and no data were collected using alternative devices such as mobile phones or commercial AI glasses. As a result, the performance of the proposed approach on other hardware platforms, which may differ substantially in camera optics, autofocus behaviour, field of view, and onboard processing capabilities, remains unknown. In addition, this work does not present a direct empirical comparison between the proposed open-vocabulary detection framework and alternative approaches, such as closed-set YOLO models trained on construction-specific datasets or large vision-language models used in commercial systems. Systematic cross-device and cross-model benchmarking represents an important direction for future research.

### Benefits of temporal majority voting

We found that a 50-frame majority voting improves accuracy and consistency by leveraging temporal redundancy in video sequences to filter out noise and occasional misclassifications. Random errors, such as those caused by motion blur, occlusions, or poor lighting, are often isolated to specific frames. By considering predictions from consecutive frames, the method smooths out these errors. Since objects typically remain visible across multiple frames, temporal redundancy ensures that even if one frame is misclassified, surrounding frames correct it. Majority voting also aggregates predictions, creating a more stable and reliable output. Instead of abrupt classification changes due to isolated errors, the final result becomes smoother and more consistent over time.

However, while increasing *K* improves stability, it also impacts inference time. Higher values of *K* require processing and storing predictions from a larger number of frames before making a final decision, which can introduce latency in real-time applications. Once *K* is large enough to cover the typical visibility duration of relevant objects, additional frames contribute little new information and mainly add delay. Very large *K* values can even cause small fluctuations in performance, as seen when the F1 score changes only by about 0.001 when *K* is above 90. In our tests, *K* = 50 provided most of the benefit while requiring ~1.7 s of video (at 30 frames per second) before issuing a final detection result. This short delay allows the system to aggregate evidence over time, while keeping detection latency low enough for practical use. Beyond this threshold, improvements become marginal, and the additional computational overhead may reduce responsiveness from the user’s perspective. Thus, selecting an appropriate *K* involves balancing detection reliability with real-time performance constraints.

### Future directions

Future improvements to the system should focus on both technical enhancements and refined detection methods. Currently, the model may still misclassify certain non-harmful objects as construction sites. Addressing this issue will involve improving the algorithm’s ability to identify features such as road lanes, which can help eliminate false positives that appear across the road. Additionally, incorporating other AI models can expand the system’s functionality and improve contextual awareness. For example, semantic segmentation models could further refine object classification by analysing road structure and distinguishing between construction barriers and regular roadside objects. Transformer-based visual language models could enhance the system’s ability to recognise environmental context and generate more accurate scene descriptions—reducing misclassifications caused by ambiguous visual features and helping the system differentiate between actual construction zones and visually similar but non-hazardous objects. Depth estimation models could also be used to estimate the distance between the user and construction obstacles, enabling the system to provide more precise navigational guidance. By adding depth information (potentially integrated with object detection and/or semantic segmentation information), the system could help users determine whether they can safely pass through a construction zone or if they need to reroute. By fine-tuning the detection process and integrating these AI advancements, the system will be better equipped to recognise and disregard objects that do not pose a hazard while improving overall detection reliability.

Another key area of advancement involves integrating real-time updates. Forging partnerships with city planning departments can facilitate the sharing of up-to-date information on active construction sites, leading to more accurate warnings and alerts. While some agencies—like the Department of Buildings in New York City—already maintain records of ongoing construction work, these datasets are often compiled passively when permits are issued or expire. Moreover, the level of impact on sidewalks is not always captured. By allowing users to record and share precise locations of obstructed walkways, the system can actively update municipal data, ensuring a live map of sidewalk construction sites. This real-time exchange of information has the potential to greatly enhance public safety and accessibility.

Finally, extensive field testing is critical to validate the system’s effectiveness in real-world scenarios. All testing so far has served as technical validation. The performance of this system with real users with blindness and low vision remains unexplored. By conducting usability studies with pBLV, we can gather valuable feedback on any remaining gaps or shortcomings in the design. This process not only helps refine the technology but also ensures that the solution genuinely meets the needs of those who may rely on it most.

## Conclusion

This study presents a novel computer vision-based system designed to detect construction sites and their associated elements, addressing a critical gap in assistive technology for pBLV. By integrating an open-vocabulary object detection model, a scaffolding-specific YOLO model, and an OCR-based construction sign recognition module, the system provides a comprehensive solution for navigating dynamic urban environments with high construction density. The system demonstrates technical feasibility and robustness, achieving high detection accuracy in controlled tests and reasonable performance in real-world scenarios. Static testing showed 100% accuracy for large, easily recognisable objects like traffic cones, barrels, and green construction walls across varying distances and angles. However, smaller or visually complex objects, such as scaffolding poles and construction signs, posed challenges due to angle-dependent visibility, motion blur, and camera limitations. Dynamic testing achieved an 87.26% accuracy rate in distinguishing construction sites from non-construction areas, though false positives from distant construction sites and irrelevant construction-related objects highlighted areas for improvement. With a 50-frame majority vote filter, the accuracy could increase to 92.0%. These findings underscore the potential of integrating advanced computer-vision techniques, such as open-vocabulary detection and OCR, to enhance accessibility for pBLV. While open-vocabulary detection shows great promise for assistive technology and construction- site monitoring, it remains prone to false detections. Complementary approaches, such as regular YOLO models or redundant null classes, are necessary to improve overall accuracy and reliability. Future advancements will focus on refining detection algorithms, incorporating real-time construction data in collaboration with municipal agencies, and conducting usability testing with pBLV to further enhance independence and safety in urban navigation.

## Figures and Tables

**Table 1.** Detection success rates at different distances and angles.

Distance/angle	0°	15°	30°	45°	60°	75°	Mean

2 m	100%	100%	100%	100%	100%	100%	100%
4 m	100%	100%	100%	100%	100%	100%	100%
6 m	86%	100%	100%	100%	100%	86%	95.3%
8 m	86%	86%	100%	86%	86%	57%	83.5%
10 m	86%	86%	57%	57%	57%	43%	64.3%
Mean	91.6%	94.4%	91.4%	88.6%	88.6%	77.2%	88.6%

**Table 2.** Confusion matrix for the dynamic testing.

Ground truth	Predicted: Positive	Predicted: Negative

Positive	3260	778
Negative	1116	9713

Rows represent ground truth labels, and columns represent predicted labels.

**Table 3.** Effect of varying majority voting window size *K* on detection performance.

*K*	Accuracy	Precision	Recall	F1 score

1	0.873	0.745	0.807	0.775
5	0.896	0.788	0.842	0.814
10	0.903	0.814	0.833	0.824
20	0.909	0.821	0.852	0.836
30	0.915	0.828	0.868	0.848
40	0.918	0.836	0.868	0.852
50	0.920	0.839	0.872	0.855
60	0.921	0.842	0.874	0.857
70	0.924	0.847	0.876	0.861
80	0.931	0.860	0.884	0.872
90	0.934	0.864	0.888	0.876
100	0.933	0.862	0.888	0.875

**Table 4.** Ablation study results: detection rate, false alarm rate, and false alarms per minute for each system configuration on the dynamic walking dataset.

Configuration	Detection rate (%)	False alarm rate (%)	False alarms/min

Full system	80.70	10.30	135.1
Minus YOLO-World	45.40	2.500	32.60
Minus scaffolding YOLO	42.00	8.900	116.2
Minus OCR	79.80	10.30	135.2
Scaffolding YOLO only	43.00	2.100	26.90
YOLO-World only	41.10	8.500	111.0
OCR only	2.400	0.500	5.900

**Table 5.** Event-based alarm summary.

Category	Count	Rate (per min)

Total alarm events	28	2.8
True positive alarms	14	1.4
False alarms (total)	14	1.4
Opposite side sites	8	0.8
Construction objects only	2	0.2
**Complete false positives**	**4**	**0.4**
False alarms by source		
YOLO-WORLD only	7	0.7
Scaffolding YOLO only	4	0.4
Joint false positives	3	0.3

The bold values in Table 5 denote complete false positives, representing alarm events in which no construction activity or relevant hazard was present. These values are highlighted to distinguish pure false alarms from context-related false alarms, such as construction on the opposite side of the street or non-hazardous construction objects.

## IMPLICATIONS FOR REHABILITATION

- **Enhanced assistive navigation:** This study developed a real-time construction site detection system to mitigate navigational hazards for individuals with blindness or low vision. By accurately identifying construction-related objects, particularly those obstructing sidewalks, this technology enhances safety and facilitates more efficient travel in urban environments.
- **Open vocabulary object detection for assistive technology:** This work explores the potential of open vocabulary zero-shot object detection for hazard identification. This approach provides valuable insights into leveraging advanced AI-driven solutions for assistive technology, enhancing adaptability and robustness in dynamic urban settings.
